# Supplementary material for: LIG-Based High-Sensitivity Multiplexed Sensing System for Simultaneous Monitoring of Metabolites and Electrolytes
Source: Sensors (Basel). 2024 Oct 29;24(21):6945. doi: 10.3390/s24216945 (PMC11548767; doi:10.3390/s24216945)
Supplement: Supplementary file 1 [file sensors-24-06945-s001.zip › sensors-3272181-supplementary.pdf]

Supplementary material

LIG-based high sensitivity multiplexed sensing system for simultaneous monitoring of metabolites and electrolytes

[1]*Sang Hyun Park*, [1]*James Jungho Pak**

[1] School of Electrical Engineering, Korea University, Seoul, Republic of Korea

*Corresponding Author: James Jungho Pak,
Current address. 145 Anam-ro, Seongbuk-gu, Korea University, Seoul, Republic of Korea, Email: pak@korea.ac.kr Phone: +82 10-7311-1858

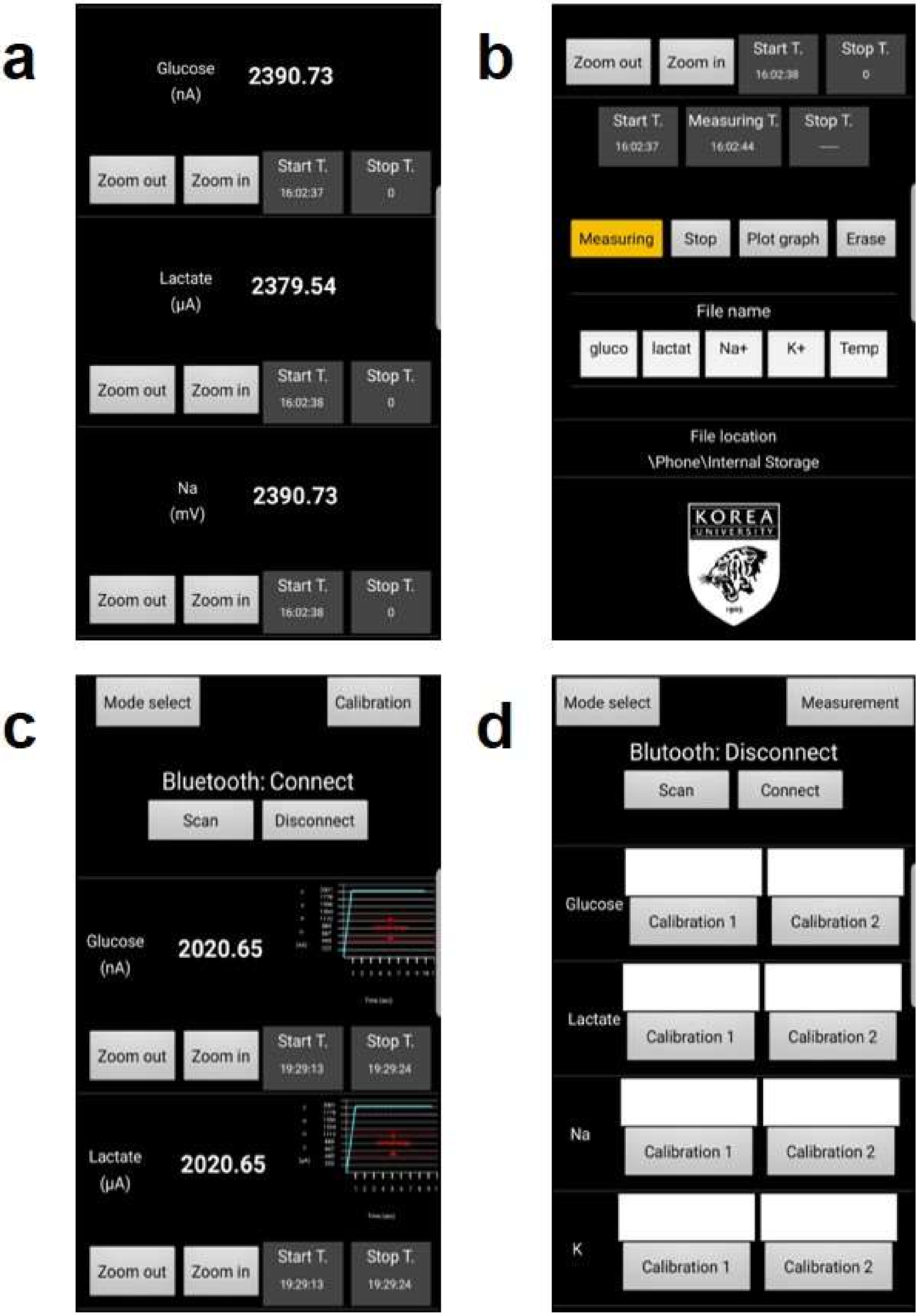

Supplementary Figure S1. (a) Smartphone screen during measurement. (b) Screen of multiplexed sensing system showing control buttons and data file name setting tabs. (c) Screen of graph created in the application after measurement is finished. (d) Calibration screen for each target analyte.

Supplementary Table S1. Concentrations of interference substances used to confirm the selectivity of each sensor.

Sensors	Glucose sensor	Lactate sensor	Na^+ sensor	K^+ sensor
Bare solution	1× PBS		DI water	
Interference solution	Acetic acid (5 mM)	Acetic acid (5 mM)	KCl (5mM)	NaCl (5mM)
	Uric acid (5 mM)	Uric acid (5 mM)	NH_4Cl (5mM)	NH_4Cl (5mM)
	Lactate (5mM)	Glucose (5mM)	$MgCl_2$ (0.5 mM)	$MgCl_2$ (0.5 mM)
	Sucrose (5mM)	Sucrose (5mM)	Ca_4Cl (0.5 mM)	Ca_4Cl (0.5 mM)
	NaCl (5mM)	NaCl (5mM)	Lactate (10mM)	Lactate (10mM)
	KCl (5mM)	KCl (5mM)	-	-

Supplementary Table S2. Concentrations of substances in artificial sweat.

	artificial sweat				
	#1	#2	#3	#4	#5
NaCl	1.169 g (10 mM)	2.338 g (20 mM)	4.676 g (40 mM)	9.352 g (80 mM)	18.704 g (160 mM)
KCl	149 mg (1 mM)	298 mg (2 mM)	596 mg (4 mM)	1.192 g (8 mM)	2.384 g (16 mM)
Glucose	0 mg (0 mM)	18 mg (50 μM)	36 mg (100 μM)	54 mg (150 μM)	72 mg (200 μM)
Lactate	149 μL (1 mM)	1.491 mL (10 mM)	2.236 mL (15 mM)	2.981 mL (20 mM)	3.726 mL (25 mM)
Urea	2.643 g (22 mM)				
Uric acid	321 mg (3 mM)				
NH_4Cl	89 mg (0.4 mM)				
$CaCl_2$	10 mg (50 μM)				
$MgCl_2$	8 mg (25 μM)				

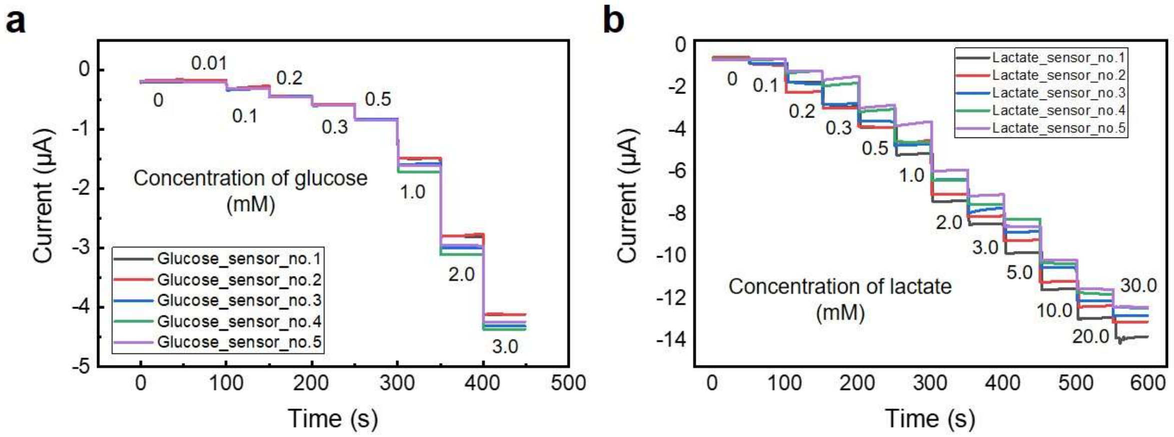

Supplementary Figure S2. Plot of (a) glucose and (b) lactate sensor's amperometric current according to measurement time.

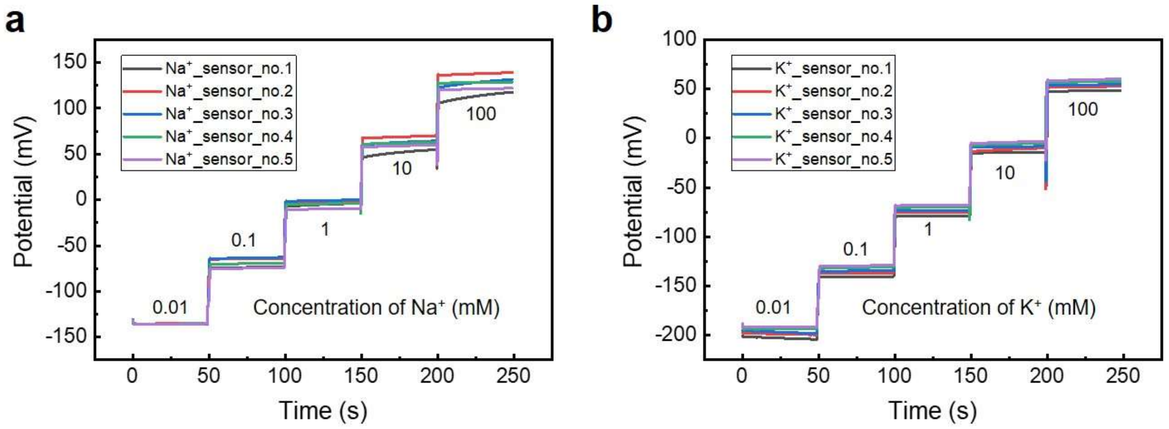

Supplementary Figure S3. Plot of a Na^+ and b K^+ sensor's potential according to measurement time.

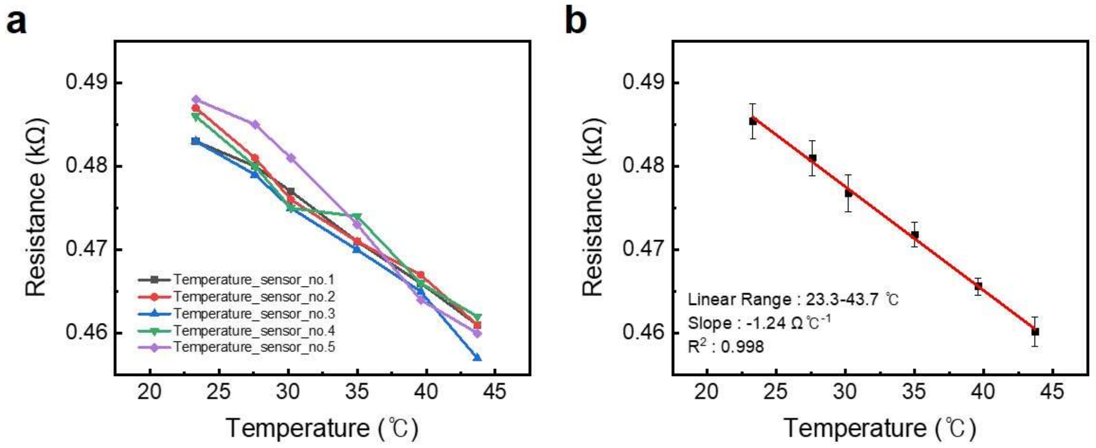

Supplementary Figure S4. (a) Plot of temperature sensor's resistance according to temperature. (b) Plot of mean and standard deviation of temperature sensor's resistance according to temperature.

Supplementary Table S3. Sensitivity and LOD of enzymatic glucose sensors.

Ref.	Structure	Sensitivity ($\mu AmM^{-1}cm^{-2}$)	LOD (μM)	Range (mM)	Applied voltage (V)
[1]	(CS-NGr-GOx)$_2$/AuE/PEDOT-PSS	237	41	0.1-1.4	−0.2
[2]	Ferrocyanide-HRP-GOx/MWCNTs/ Stainless-steel Pin	1.44	30	0.05-1	−0.2
[3]	HRP-GOx-GA/ KB/GCE	330	2	0.005-1.5	−0.1
[4]	GOx /Au-PtNFs	84.67	10	0.01-4	0
[5]	Nafion/(GOx/MWCNTs-Cysc)$_2$/GCE	0.096	8	0.1-1	−0.05
[6,7]	TCNFs-GOx/GCE	628.82	3.7	0.013-10.5	−0.55
[8]	biot-GOx/RuNPs/MWCNTs-Av/GCE	2.6	3.3	0.02-1.23	−0.05
[9]	GOx/CdS–ZnS-QDs/PGE	11.5	3	0.01-1	−0.5
[10]	$CaTiO_3$NPs-GOx-CS/GCE	14.1	2.3	0.007-1.49	−0.45
[11]	GOx-CS/TiO_2NRs/GMFE	18.6	2.2	1.5-	−0.5
[12]	GOx/IL-CHO/ERGO/SPCE	17.7	17	0.05-2.4	−0.45
[13]	GOx/HNF–TiO2/GCE	32.6	0.8	0.002-3.17	−0.45
[14]	GA/GOx-BSA/ Fe_3O_4-PB/GCE	32	0.5	0.005-1.2	−0.15
[15]	Naf /GA/ GOx / GNS-IL-AuNRs (sol-gel)/ AuNFs/GCE	0.0064	0.38	0.001-0.764	−0.2
[16]	GOx/GNS-PEI-AuNPs/AuE/	93	0.32	0.001-0.1	−0.35
[17]	Nafion/GO_x/Au-ZnO/rGO/ITO(UV)	10.93	0.2	0-9.5	0.8
This work	GOx/PdCu/LIG	168.15	0.191	0-3	0

Supplementary Table S4. Sensitivity and LOD of enzymatic lactate sensors.

Ref.	Structure	Sensitivity (μAmM^{-1}cm^{-2})	LOD (μM)	Range (mM)	Applied voltage (V)
[18]	LOx-microband-SPCE	0.0036	289	1-10	0.4
[19]	Lox/BSA/HRP/CS/FcMe/ MWCNT/SPBGE	3.417	22.6	0.0304-0.2439	−0.2
[20]	LOx-FSM8.0/Nafion/CoPCSPC	4.54	18	0.0183-1.5	0.45
[21]	LOx/PtNp-CNF-PDDA/SPCEs	36.8	11	0.025-1.5	0.5
[22]	LOx-SPCE/graphite/HRP	0.87	10	0.01-0.2	0
[23]	GCNF–PEI–GA–LOx–Gly-SPCE/PtNps	41.3	6.9	0.01-20	0.3
[24]	PDDA/LOx/ZnO/MWCNT	7.3	160	0.2-2.0	0.4
[25]	LOx/N-CNT/GC	40	4.1	0.014-0.325	−0.23
[26]	LOx/AuNP/CNT/Gr/Pt	35.3	2.3	0.05-100	0.2
[27]	Nafion/LOx/ZnO/Au	28	1.2	0.0036-0.6	0.8
[28]	LOx-SPCE/BSA/Fc-HRP/MWCNT/PS	1.1688	0.5	0.001-0.0312	0.65
This work	Lox/PdCu/LIG	872.1	0.167	0-0.5	0

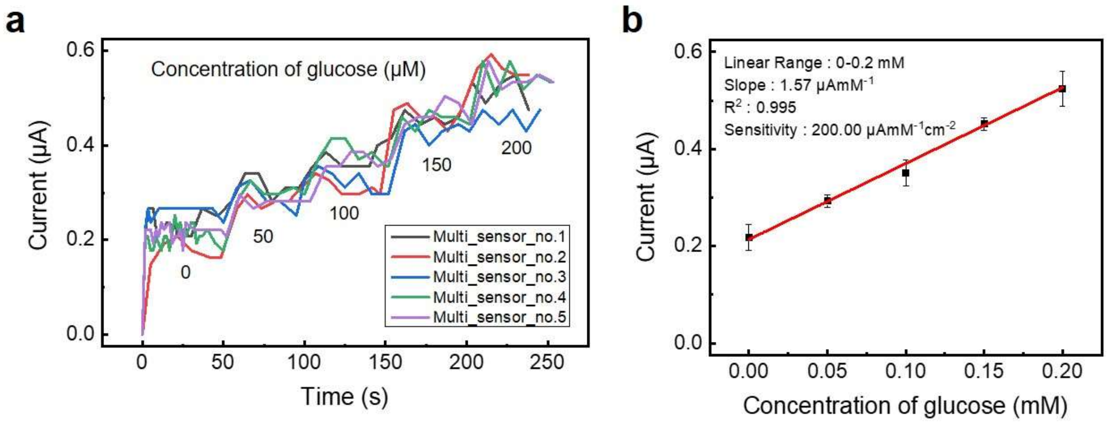

Supplementary Figure S5. a Plot of glucose sensor and multiplexed sensing system's amperometric current according to measurement time. b Plot of mean and standard deviation of glucose sensor and multiplexed sensing system's amperometric current according to glucose concentration.

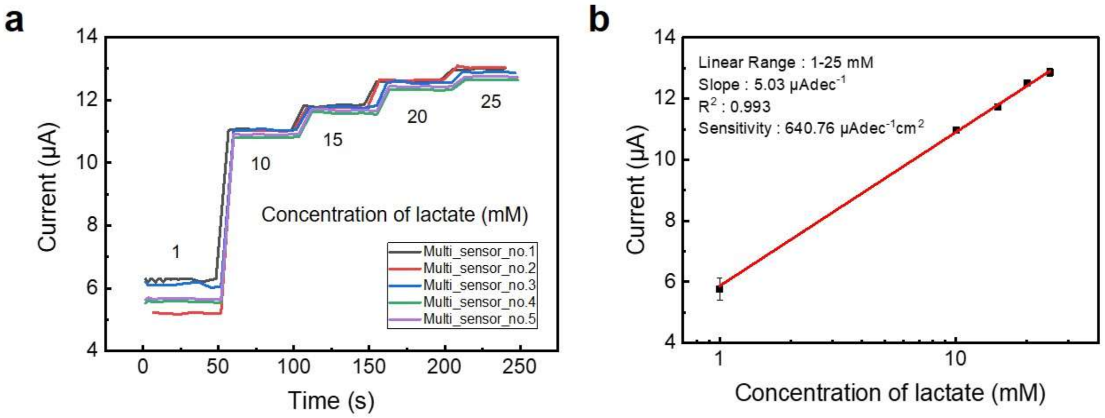

Supplementary Figure S6. (a) Plot of lactate sensor and multiplexed sensing system's amperometric current according to measurement time. (b) Plot of mean and standard deviation of lactate sensor and multiplexed sensing system's amperometric current according to lactate concentration.

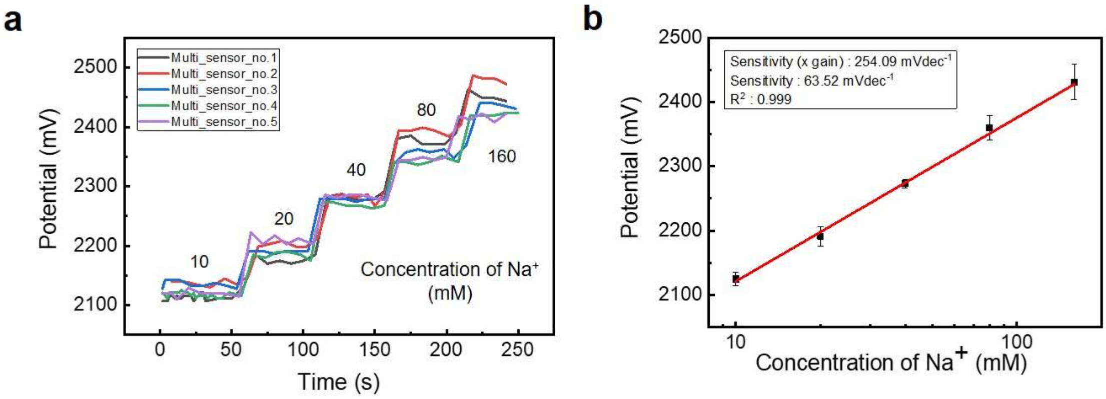

Supplementary Figure S7. (a) Plot of Na^+ sensor and multiplexed sensing system's potential according to measurement time. (b) Plot of mean and standard deviation of Na^+ sensor and multiplexed sensing system's potential according to Na^+ concentration.

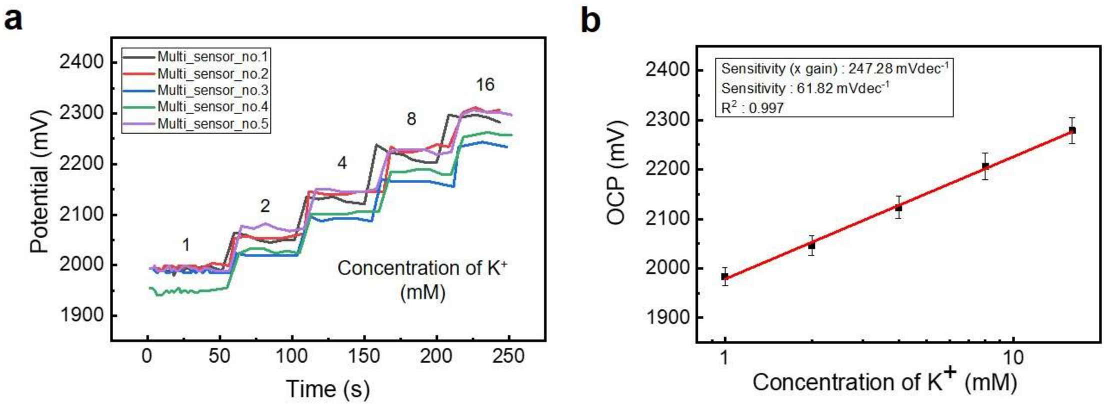

Supplementary Figure S8. (a) Plot of K^+ sensor and multiplexed sensing system's potential according to measurement time. (b) Plot of mean and standard deviation of K^+ sensor and multiplexed sensing system's potential according to K^+ concentration.

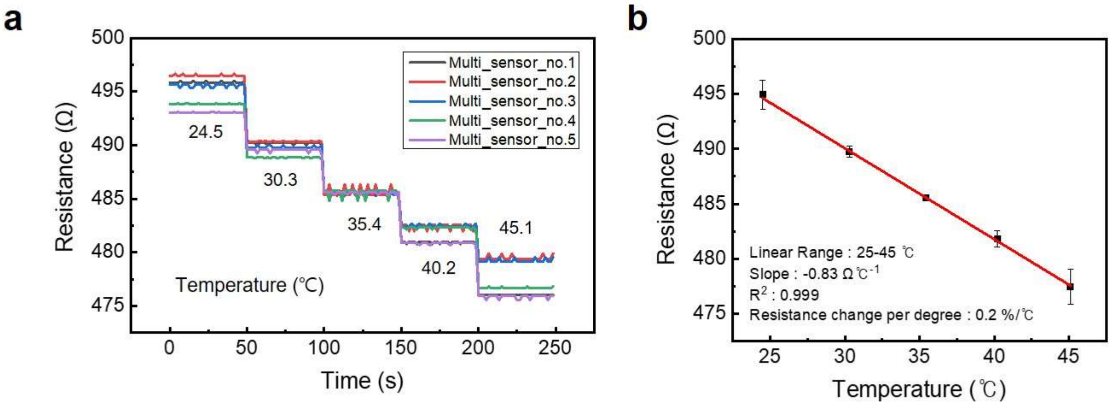

Supplementary Figure S9. (a) Plot of temperature sensor and multiplexed sensing system's resistance according to measurement time. (b) Plot of mean and standard deviation of temperature sensor and multiplexed sensing system's resistance according to temperature.

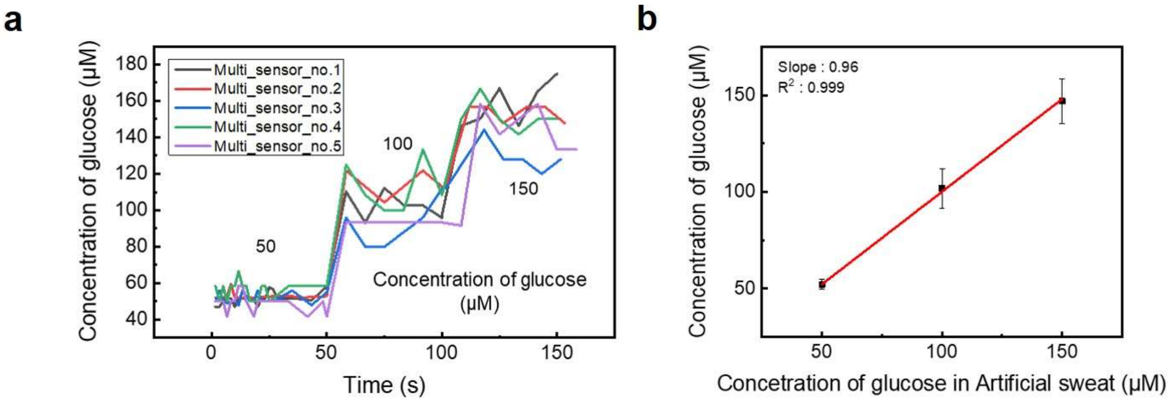

Supplementary Figure S10. (a) Plot of calibrated glucose sensor and multiplexed sensing system's concentration according to measurement time. (b) Plot of mean and standard deviation of calibrated glucose sensor and multiplexed sensing system's concentration according to glucose concentration of artificial sweat.

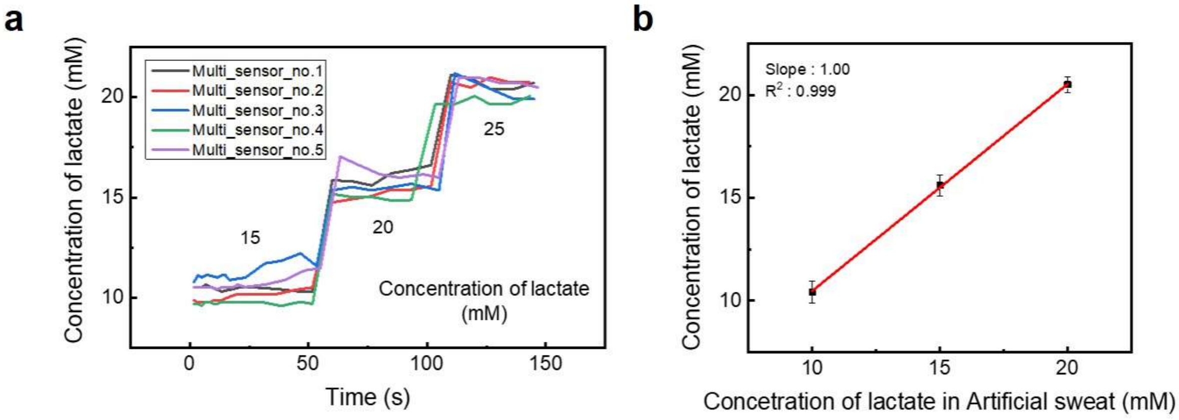

Supplementary Figure S11. (a) Plot of calibrated lactate sensor and multiplexed sensing system's concentration according to measurement time. (b) Plot of mean and standard deviation of calibrated lactate sensor and multiplexed sensing system's concentration according to lactate concentration of artificial sweat.

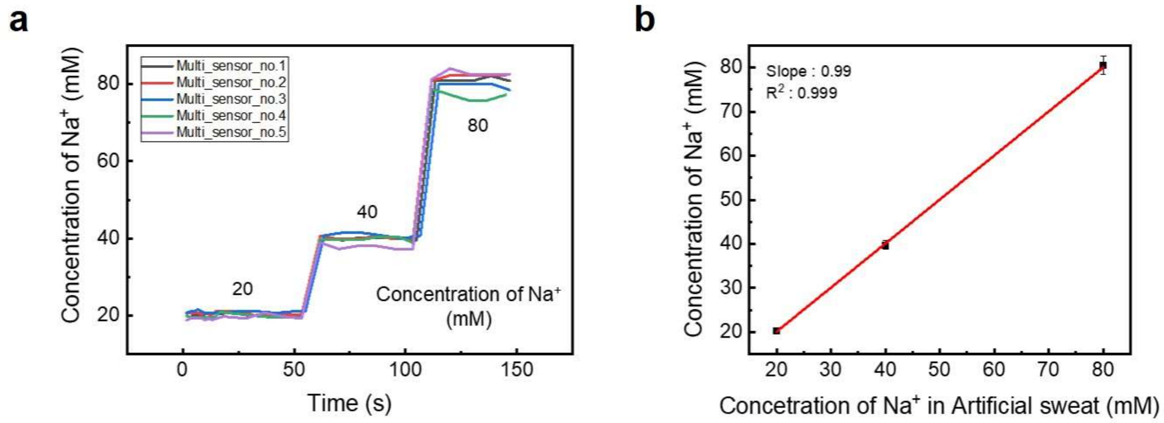

Supplementary Figure S12. (a) Na^+ concentration vs. time plot and (b) mean and standard deviation plot acquired by calibrated Na^+ sensor and multiplexed sensing system.

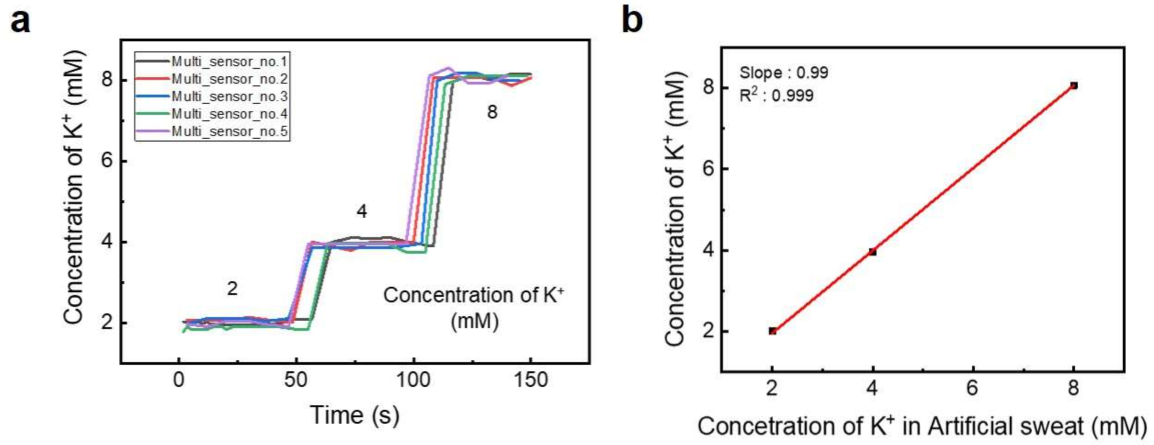

Supplementary Figure S13. (a) K^+ concentration vs. time plot and (b) mean and standard deviation plot acquired by calibrated K^+ sensor and multiplexed sensing system.

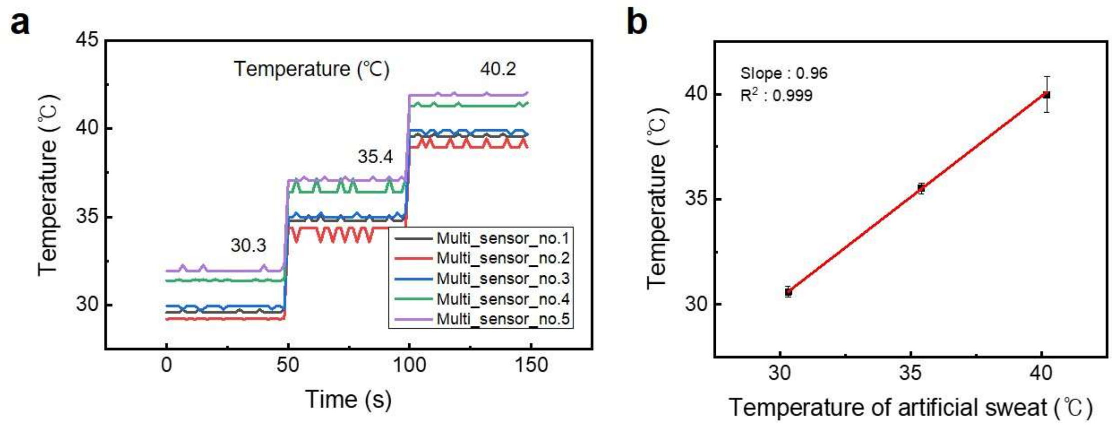

Supplementary Figure S14. (a) Temperature of artificial sweat vs. time plot and (b) mean and standard deviation plot acquired by calibrated temperature sensor and multiplexed sensing system.

References

1. David, M.; Barsan, M.M.; Brett, C.M.A.; Florescu, M. Improved Glucose Label-Free Biosensor with Layer-by-Layer Architecture and Conducting Polymer Poly(3,4-Ethylenedioxythiophene). *Sensors Actuators, B Chem.* **2018**, *255*, 3227–3234, doi:10.1016/j.snb.2017.09.149.

2. Rama, E.C.; Costa-García, A.; Fernández-Abedul, M.T. Pin-Based Electrochemical Glucose Sensor with Multiplexing Possibilities. *Biosens. Bioelectron.* **2017**, *88*, 34–40, doi:10.1016/j.bios.2016.06.068.
3. Xia, H.Q.; Kitazumi, Y.; Shirai, O.; Kano, K. Direct Electron Transfer-Type Bioelectrocatalysis of Peroxidase at Mesoporous Carbon Electrodes and Its Application for Glucose Determination Based on Bienzyme System. *Anal. Sci.* **2017**, *33*, 839–844, doi:10.2116/analsci.33.839.
4. Zhu, Q.; Liang, B.; Liang, Y.; Ji, L.; Cai, Y.; Wu, K.; Tu, T.; Ren, H.; Huang, B.; Wei, J.; et al. 3D Bimetallic Au/Pt Nanoflowers Decorated Needle-Type Microelectrode for Direct in Situ Monitoring of ATP Secreted from Living Cells. *Biosens. Bioelectron.* **2020**, *153*, doi:10.1016/j.bios.2020.112019.
5. Eguílaz, M.; Venegas, C.J.; Gutiérrez, A.; Rivas, G.A.; Bollo, S. Carbon Nanotubes Non-Covalently Functionalized with Cytochrome c: A New Bioanalytical Platform for Building Bienzymatic Biosensors. *Microchem. J.* **2016**, *128*, 161–165, doi:10.1016/j.microc.2016.04.018.
6. Manoj, D.; Theyagarajan, K.; Saravanakumar, D.; Senthilkumar, S.; Thenmozhi, K. Aldehyde Functionalized Ionic Liquid on Electrochemically Reduced Graphene Oxide as a Versatile Platform for Covalent Immobilization of Biomolecules and Biosensing. *Biosens. Bioelectron.* **2018**, *103*, 104–112, doi:10.1016/j.bios.2017.12.030.
7. Guo, Q.; Liu, L.; Wu, T.; Wang, Q.; Wang, H.; Liang, J.; Chen, S. Flexible and Conductive Titanium Carbide–Carbon Nanofibers for High-Performance Glucose Biosensing. *Electrochim. Acta* **2018**, *281*, 517–524, doi:10.1016/j.electacta.2018.05.181.
8. Gallay, P.; Eguílaz, M.; Rivas, G. Designing Electrochemical Interfaces Based on Nanohybrids of Avidin Functionalized-Carbon Nanotubes and Ruthenium Nanoparticles as Peroxidase-like Nanozyme with Supramolecular Recognition Properties for Site-Specific Anchoring of Biotinylated Residues. *Biosens. Bioelectron.* **2020**, *148*, 111764, doi:10.1016/j.bios.2019.111764.
9. Saɫlam, Ö.; KIzIlkaya, B.; Uysal, H.; Dilgin, Y. Biosensing of Glucose in Flow Injection Analysis System Based on Glucose Oxidase-Quantum Dot Modified Pencil Graphite Electrode. *Talanta* **2016**, *147*, 315–321, doi:10.1016/j.talanta.2015.09.050.
10. Wang, L.; Li, J.; Feng, M.; Min, L.; Yang, J.; Yu, S.; Zhang, Y.; Hu, X.; Yang, Z. Perovskite-Type Calcium Titanate Nanoparticles as Novel Matrix for Designing Sensitive Electrochemical Biosensing. *Biosens. Bioelectron.* **2017**, *96*, 220–226, doi:10.1016/j.bios.2017.05.004.
11. Zhang, J.; Yu, X.; Guo, W.; Qiu, J.; Mou, X.; Li, A.; Liu, H. Construction of Titanium Dioxide Nanorod/Graphite Microfiber Hybrid Electrodes for a High Performance Electrochemical Glucose Biosensor. *Nanoscale* **2016**, *8*, 9382–9389, doi:10.1039/c6nr01360k.
12. Wang, Z.X.; Wang, J.Y.; Yu, X.H.; Kong, F.Y.; Wang, W.J.; Lv, W.X.; Ge, L.; Wang, W. Synergistic Contributions by Decreasing Overpotential and Enhancing Electrocatalytic Reduction in ONPCNRs/SWCNTs Nanocomposite for Highly Sensitive Nonenzymatic Detection of Hydrogen Peroxide. *Sensors Actuators, B Chem.* **2017**, *246*, 726–733, doi:10.1016/j.snb.2017.02.102.
13. Guo, Q.; Liu, L.; Zhang, M.; Hou, H.; Song, Y.; Wang, H.; Zhong, B.; Wang, L. Hierarchically Mesostructured Porous TiO2 Hollow Nanofibers for High Performance Glucose Biosensing. *Biosens. Bioelectron.* **2017**, *92*, 654–660, doi:10.1016/j.bios.2016.10.036.
14. Jomma, E.Y.; Ding, S.N. One-Pot Hydrothermal Synthesis of Magnetite Prussian Blue Nano-Composites and Their Application to Fabricate Glucose Biosensor. *Sensors (Switzerland)* **2016**, *16*, doi:10.3390/s16020243.
15. Tang, H.; Cai, D.; Ren, T.; Xiong, P.; Liu, Y.; Gu, H.; Shi, G. Fabrication of a Low Background Signal Glucose Biosensor with 3D Network Materials as the Electrocatalyst. *Anal. Biochem.* **2019**, *567*, 63–71, doi:10.1016/j.ab.2018.12.012.
16. Rafighi, P.; Tavahodi, M.; Haghighi, B. Fabrication of a Third-Generation Glucose Biosensor Using Graphene-Polyethyleneimine-Gold Nanoparticles Hybrid. *Sensors Actuators, B Chem.* **2016**, *232*, 454–461, doi:10.1016/j.snb.2016.03.147.

17. Zhou, F.; Jing, W.; Liu, S.; Mao, Q.; Xu, Y.; Han, F.; Wei, Z.; Jiang, Z. Electrodeposition of Gold Nanoparticles on ZnO Nanorods for Improved Performance of Enzymatic Glucose Sensors. *Mater. Sci. Semicond. Process.* **2020**, *105*, 104708, doi:10.1016/j.mssp.2019.104708.
18. Rawson, F.J.; Purcell, W.M.; Xu, J.; Pemberton, R.M.; Fielden, P.R.; Biddle, N.; Hart, J.P. A Microband Lactate Biosensor Fabricated Using a Water-Based Screen-Printed Carbon Ink. *Talanta* **2009**, *77*, 1149–1154, doi:10.1016/j.talanta.2008.08.020.
19. Hernández-Ibáñez, N.; García-Cruz, L.; Montiel, V.; Foster, C.W.; Banks, C.E.; Iniesta, J. Electrochemical Lactate Biosensor Based upon Chitosan/Carbon Nanotubes Modified Screen-Printed Graphite Electrodes for the Determination of Lactate in Embryonic Cell Cultures. *Biosens. Bioelectron.* **2016**, *77*, 1168–1174, doi:10.1016/j.bios.2015.11.005.
20. Shimomura, T.; Sumiya, T.; Ono, M.; Ito, T.; Hanaoka, T. aki Amperometric L-Lactate Biosensor Based on Screen-Printed Carbon Electrode Containing Cobalt Phthalocyanine, Coated with Lactate Oxidase-Mesoporous Silica Conjugate Layer. *Anal. Chim. Acta* **2012**, *714*, 114–120, doi:10.1016/j.aca.2011.11.053.
21. Lamas-Ardisana, P.J.; Loaiza, O.A.; Añorga, L.; Jubete, E.; Borghei, M.; Ruiz, V.; Ochoteco, E.; Cabañero, G.; Grande, H.J. Disposable Amperometric Biosensor Based on Lactate Oxidase Immobilised on Platinum Nanoparticle-Decorated Carbon Nanofiber and Poly(Diallyldimethylammonium Chloride) Films. *Biosens. Bioelectron.* **2014**, *56*, 345–351, doi:10.1016/j.bios.2014.01.047.
22. Ghamouss, F.; Ledru, S.; Ruillé, N.; Lantier, F.; Boujtita, M. Bulk-Modified Modified Screen-Printing Carbon Electrodes with Both Lactate Oxidase (LOD) and Horseradish Peroxide (HRP) for the Determination of l-Lactate in Flow Injection Analysis Mode. *Anal. Chim. Acta* **2006**, *570*, 158–164, doi:10.1016/j.aca.2006.04.022.
23. Loaiza, O.A.; Lamas-Ardisana, P.J.; Añorga, L.; Jubete, E.; Ruiz, V.; Borghei, M.; Cabañero, G.; Grande, H.J. Graphitized Carbon Nanofiber-Pt Nanoparticle Hybrids as Sensitive Tool for Preparation of Screen Printing Biosensors. Detection of Lactate in Wines and Ciders. *Bioelectrochemistry* 2015, *101*, 58–65.
24. Wang, Y.T.; Bao, Y.J.; Lou, L.; Li, J.J.; Du, W.J.; Zhu, Z.Q.; Peng, H.; Zhu, J.Z. A Novel L-Lactate Sensor Based on Enzyme Electrode Modified with ZnO Nanoparticles and Multiwall Carbon Nanotubes. *Proc. IEEE Sensors* **2010**, 33–37, doi:10.1109/ICSENS.2010.5690980.
25. Goran, J.M.; Lyon, J.L.; Stevenson, K.J. Amperometric Detection of L-Lactate Using Nitrogen-Doped Carbon Nanotubes Modified with Lactate Oxidase. *Anal. Chem.* **2011**, *83*, 8123–8129, doi:10.1021/ac2016272.
26. Hashemzadeh, S.; Omidi, Y.; Rafii-Tabar, H. Amperometric Lactate Nanobiosensor Based on Reduced Graphene Oxide, Carbon Nanotube and Gold Nanoparticle Nanocomposite. *Microchim. Acta* **2019**, *186*, doi:10.1007/s00604-019-3791-0.
27. Lei, Y.; Luo, N.; Yan, X.; Zhao, Y.; Zhang, G.; Zhang, Y. A Highly Sensitive Electrochemical Biosensor Based on Zinc Oxide Nanotetrapods for L-Lactic Acid Detection. *Nanoscale* **2012**, *4*, 3438–3443, doi:10.1039/c2nr30334e.
28. Pérez, S.; Fàbregas, E. Amperometric Bienzymatic Biosensor for L-Lactate Analysis in Wine and Beer Samples. *Analyst* **2012**, *137*, 3854–3861, doi:10.1039/c2an35227c.
